# Role of the NLRP3 inflammasome in cancer

**DOI:** 10.1186/s12943-018-0900-3

**Published:** 2018-11-17

**Authors:** Maryam Moossavi, Negin Parsamanesh, Afsane Bahrami, Stephen L. Atkin, Amirhossein Sahebkar

**Affiliations:** 1 Student Research Committee, Birjand University of Medical Sciences, Birjand, Iran; 2 Cellular and Molecular Research Center, Birjand University of Medical Sciences, Birjand, Iran; 3 Weill Cornell Medicine Qatar, Education City, PO Box 24144, Doha, Qatar; 4 Biotechnology Research Center, Pharmaceutical Technology Institute, Mashhad University of Medical Sciences, Mashhad, Iran; 5 Neurogenic Inflammation Research Center, Mashhad University of Medical Sciences, Mashhad, Iran; 6 School of Pharmacy, Mashhad University of Medical Sciences, Mashhad, Iran

**Keywords:** Nod-like receptor protein 3, Caspase-1, Interleukin-1β, Apoptosis-associated speck-like protein

## Abstract

Inflammasomes are large intracellular multi-protein signalling complexes that are formed in the cytosolic compartment as an inflammatory immune response to endogenous danger signals. The formation of the inflammasome enables activation of an inflammatory protease caspase-1, pyroptosis initiation with the subsequent cleaving of the pro-inflammatory cytokines interleukin (IL)-1β and proIL-18 to produce active forms. The inflammasome complex consists of a Nod-like receptor (NLR), the adapter apoptosis-associated speck-like (ASC) protein, and Caspase-1. Dysregulation of NLRP3 inflammasome activation is involved tumor pathogenesis, although its role in cancer development and progression remains controversial due to the inconsistent findings described. In this review, we summarize the current knowledge on the contribution of the NLRP3 inflammasome on potential cancer promotion and therapy.

## Background

The immune system identifies and eradicates pathogens through the cooperation of the native (innate) immune system and the acquired immune system [1]. The native immune system acts as the initial line of defense that is implemented in the presence of cell-derived damage associated molecular patterns (DAMPs) with or without the presence of infection [2]. These multifunctional molecules include heat shock proteins (HSPs), messenger RNA single strand RNA (ssRNA), and small fragments of extracellular matrix that are released into the extracellular environment following tissue and cellular injury [3]. Furthermore, the natural immune system detects pathogen associated molecular patterns (PAMPs) [2] derived from pathogens via pattern recognition receptors (PRRs) expressed by the cells of the innate immune system that recognize the microorganisms at the site of infection, and present the antigens to the acquired immune system [4, 5]. In 2002, the first PRR (inflammasome) was discovered [6–8] following which, various inflammasomes have been identified comprising NLRP1, NLRP2, NLRP3, absent in melanoma 2(AIM2) and NLRC4 [9]. Among them NLRP3 inflammasome is the most well described as pyrin domain containing protein 3 [10].

## Classification of pattern recognition receptors (PRRs)

PRRs can be sub-classified into two main categories based on their sub-cellular localization.

i. The first category located in the plasma membrane (transmembrane proteins) and endosomes includes Toll-like receptors (TLRs) and C-type lectin receptors (CLRs) which can recognize extracellular PAMPs and DAMPs.
ii. The second category of PRRs inhibits in intracellular partitions and involves the retinoic acid-inducible gene, RIG-I-like receptor (RLRs), AIM2-like receptor (ALRs), nucleotide-binding and oligomerization (NOD) domain like receptors (NLRs) and cytosolic sensor cyclic GMP-AMP (cGAMP) synthase (cGAS) [11, 12].

## Structure of the NLR family

**Fig. 1:**
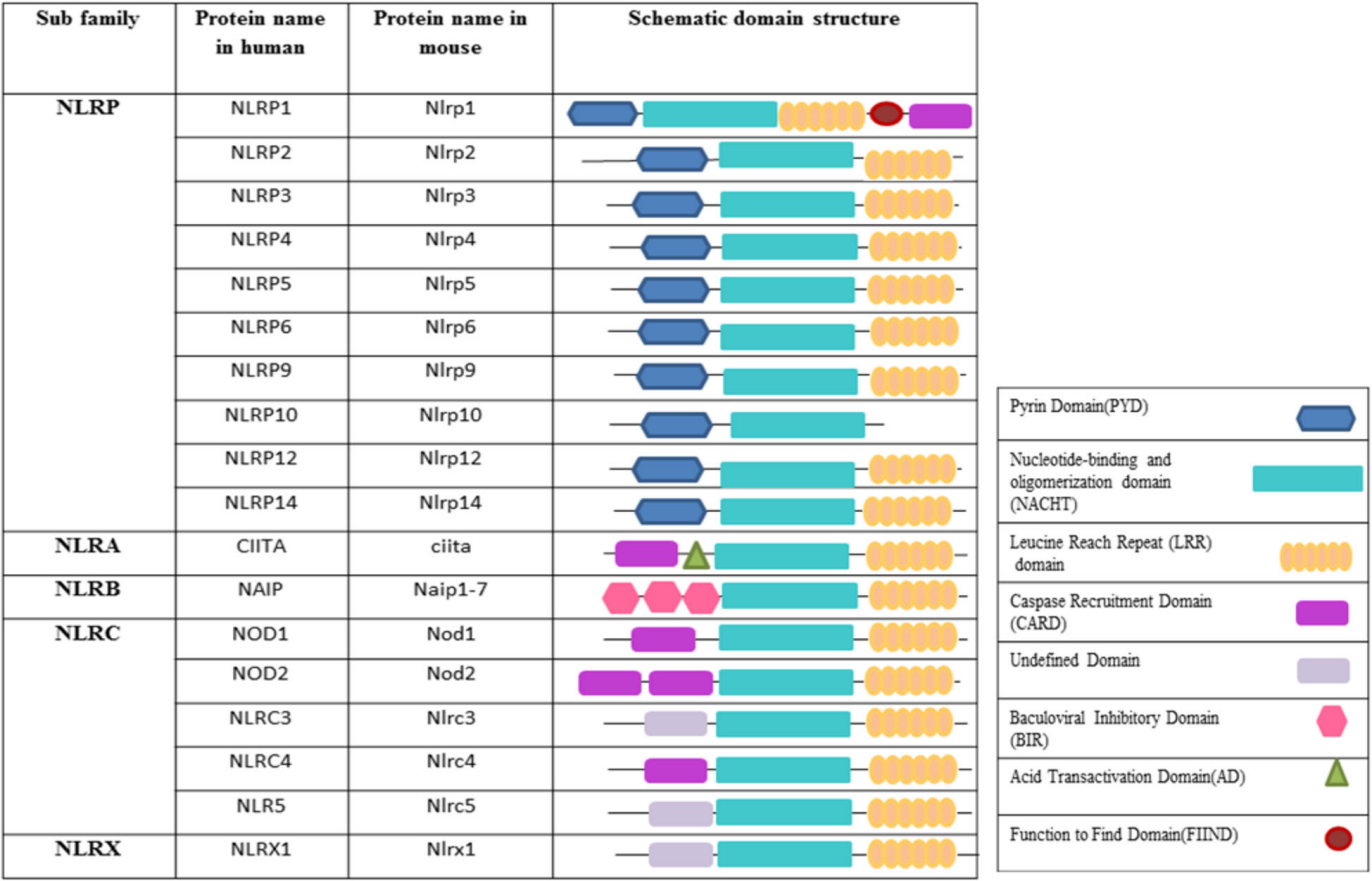
Schematic diagram of NLR gene family

All NLR family members that are located in the second category of PRRs have a main nucleotide binding domain (NBD); however, a C-terminal with leucine-rich repeats is observed in most and some of the NLR members have an N-terminal domain as well [13] (Fig. 1). These members can be sub-grouped based on the N terminal domain: a) NLRP that contain pyrin, and b) NLRC that contains the caspase activation and recruitment domain (CARD) [14]. The NLRs are derived from 22 human genes [15]. Certain types of NLRs, NLRP1, NLRP3 and NLRC4, can establish large cytosolic protein complexes (possibly hexamers or heptamers) named inflammasomes, which are responsible for the initiation cleavage and activation of procaspase-1 in human and procaspase-11 in mice, which eventually give rise to the proteolytic activation of pro-interleukin(IL)-1β and pro-IL-18 cytokines [16].

### Inflammasome history

In 2002, Tschopp et al. applied the term inflammasome to define a protein complex that mediated the stimulation of inflammatory caspases [17]. Inflammasomes are high molecular weight protein complexes activated through various pathogen infections [18] or cellular and physiological stresses that provoke a rapid release of proinflammatory cytokines that recruit native immune cells for defence against intruders [14]. Hence, impairment in the regulation of inflammasome function is associated with tumor development [19, 20], autoimmune disorders [21, 22], and neurodegenerative diseases [23, 24].

### Inflammasome oligomerization

Oligomerization of five receptor proteins in the NLR family, NLRP1, NLRP3 and NLRC4, as well as AIM2 and pyrin are requisite for inflammasome formation and activation of cysteine protease procaspase-1 [25, 26]. Active caspase-1 results in proIL-1β and proIL-18 formation of biologically active cytokines [27]. The active form of IL-1β is a powerful pro-inflammatory cytokine that recruits the native immune cells to the infected site while the active form of IL-18 is essential for interferon-γ (IFN-γ) production, augments the activity of natural killer (NK) cells and T cells [18]. Moreover, the active form of caspase-1 stimulates pyroptosis that is an inflammatory type of programmed cell death and happens most commonly with intracellular pathogen infection [18].

### The NLRP3 inflammasome complex

Nod-like receptor protein 3 (NLRP3) is one of the most characterized of the inflammasomes that belongs to the NLR protein family and contains 22 members in the human [15, 16]. NLRP3 reacts to a wide range of inflammatory infectious and endogenous ligands such as PAMPs and/or DAMPs; therefore, the dysregulation in the function of NLRP3 is associated with the pathogenesis of several inflammatory diseases [14, 28–30]. This protein complex consists of three components including a) NLRP3 scaffold, b) PYCARD (PYD And CARD Domain) adaptor, frequently referred to as apoptosis-associated speck-like protein (ASC), which functions as a caspase-1 activator and c) the third component is caspase-1 [18].

### NLRP3 inflammasome complex activation

**Fig. 2:**
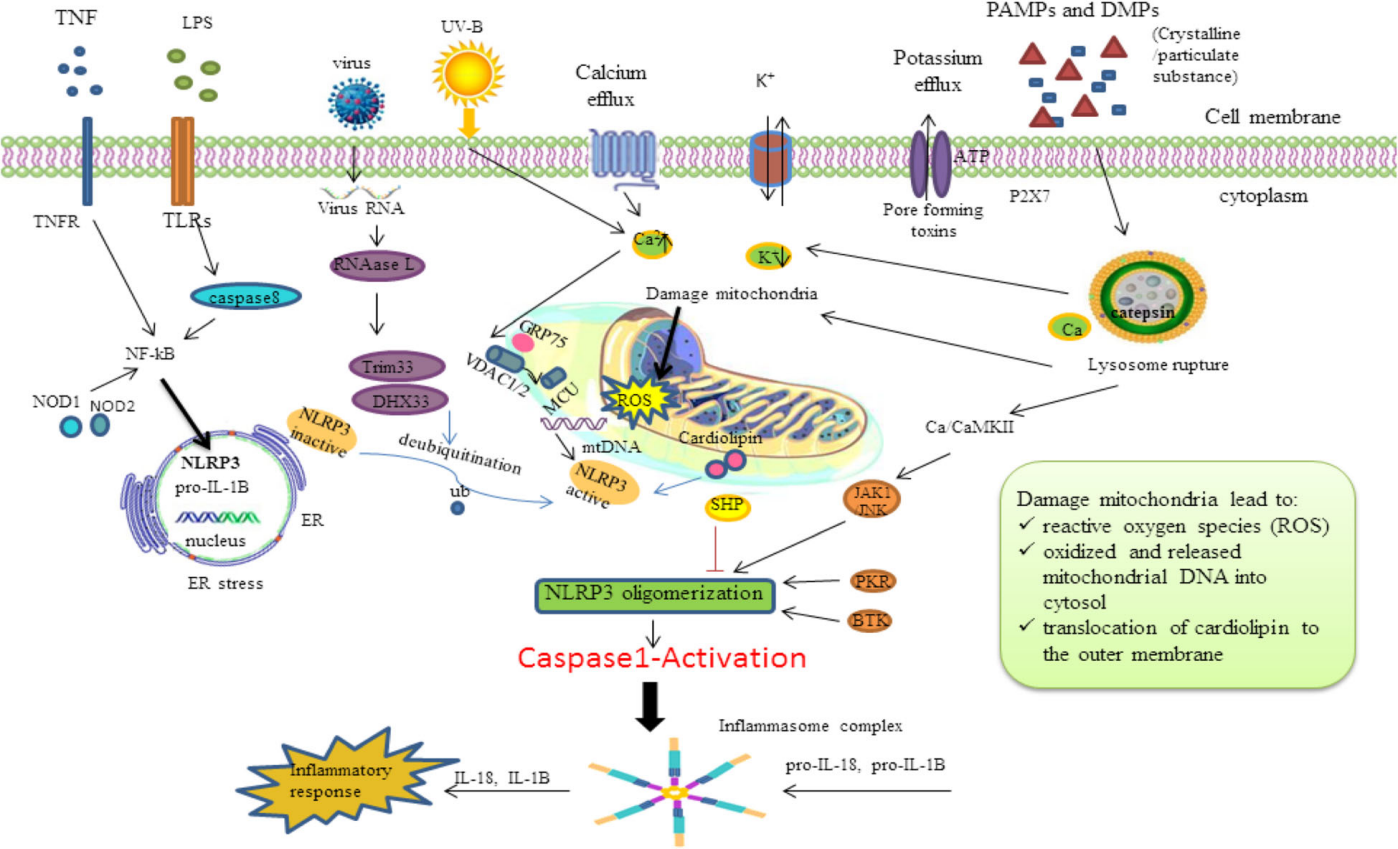
Activation and signaling of NLRP3 inflammasome. BTK(Bruton’s tyrosine kinase), CaMKII (Calcium/calmodulin-dependent protein kinase II), DAMPs(damage associated molecular patterns), DHX33(DEAH-box helicase 33), ER(endoplasmic reticulum), IL(Interleukin), JAK1(Janus family of protein tyrosine kinases), LPS(Lipopolysaccharide), MCU(mitochondrial Ca 2+ uniporter), Nuclear factor-κB (NF-κB), NLRP3(NLR family, pyrin domain containing 3), NOD(nucleotide-binding and oligomerization), PAMPs(pathogen associated molecular patterns), PKR(protein kinase R), SHP(small heterodimer partner (SHP), TNF (tumor necrosis factor), Trim33(Tripartite motif-containing protein 33), VDAC1/2(voltage-dependent anion-selective channel 1/2)

The NLRP3 complex is mainly express in immune cells notably antigen presenting cells (APCs) and inflammatory cells after the inflammatory stimulatory trigger, which comprises macrophages (a potent APC), dendritic cells (DC), neutrophils in the spleen and monocytes [31]. The two hit hypothesis has been proposed for NLRP3 activation [9]. The initial hit is when TLR is auto-phosphorylated by exposure to PAMPs and/or DAMPs and resulting in nuclear factor-κB (NF-κB) activation. This nuclear factor stimulates transcription and the expression of NLRP3 inflammasome components, proIL-1β, and proIL-18, which after translocation from a nuclear to cytoplasmic location remain inactive until the second hit [32] (Fig. 2). This hypothesis is frequently evaluated in vitro via lipopolysaccharide (LPS) [33]. The second hit assists the oligomerzation of the inactive inflammasome complex (NLRP3, ASC and caspase-1), which contributes to activation, maturation and up-regulation of IL-1β and IL-18 [9, 34]. Several models have been suggested to express the key mechanisms of the second hit of inflammasome activation that are described in detail.

1. Potassium ion efflux (a common inflammasome activator) is an essential factor for the assembly and up-regulation of NLRP3 complexes induced by the agonist adenosine triphosphate (ATP) [35]. The potassium ion efflux occurs through a purogenic P2X7-ATP dependent pore that recruits a pannexin-1 hemi-channel. This process allows extracellular NLRP3 agonists to enter the cytosol and engage the NLRP3 protein complex and triggers IL-1β secretion by the inflammasome [36, 37]. In accord with this model, several studies have shown that an elevated concentration of extracellular potassium prevents NLRP3 complex activation whilst a reduced cytoplasmic potassium concentration initiates the NLRP3 inflammasome activation, even in a cell free system [38]. Nevertheless, the molecular pathway between reduced levels of cytosolic potassium and NLRP3 activation requires further clarification.
2. Calcium (Ca+ 2) flux: cytoplasmic and endoplasmic reticulum (the major intracellular Ca2+ reservoir) Ca^2+^ flux seems to be involved in NLRP3 inflammasome activation [39–42]. Calcium flux is induced by several stimuli such as ATP [41]. It is important to mention that blockade of Ca^2+^ signalling prohibits inflammasome activation [43]. A pharmacological study showed that blockade of the inositol 1,4,5-trisphosphate(IP3) receptor, the intracellular calcium release channel on the ER, decreases Ca^2+^ flux and consequently inhibited NLRP3 activation [40], whilst adding calcium ions to RPMI medium results in potassium efflux and NLRP3 inflammasome activation [38]. However, Katsnelson et al. compared the combination of reduced cytosolic potassium concentrations with increased cytosolic Ca^2+^ in NLRP3 inflammasome activation and identified that Ca^2+^ is chiefly unnecessary for NLRP3 activation [43]; hence, the role of calcium signalling in NLRP3 complex activation remains unclear.
3. Mitochondrial dysfunction: following Ca+ 2 flux into the mitochondrial matrix and Ca+ 2 overload, mitochondrial dysfunction occurs that may support NLRP3 complex formation [41]. Mitochondrial perturbation signals, result in the production of reactive oxygen species (ROS), oxidized and released mitochondrial DNA into the cytosol, and/or translocation of a specific mitochondrial phospholipid, cardiolipin, to the outer membrane that facilitates the attachment of the LRRs of NLRP3, and that are also thought to mediate NLRP3 activation [25, 44–46]. In accord with this, PAMPs and DAMPs as well as the NLRP3 agonist ATP, activate the production of ROS that trigger the NLRP3 complex formation through damage to NADPH oxidase and other mitochondrial oxidative systems [39, 47, 48]. Cardiolipin directly activates the NLRP3 inflammasome in a pathway independent of ROS cooperation [45]; however, the molecular pathway involved in cardiolipin function for the activation of NLRP3 inflammasome complex activation requires further clarification.
4. Cytosolic release of lysosomal cathepsin-B: In this model, phagocytosis of environmental particles appears to activate the NLRP3 complex that form crystalline structures when engulfed by phagocytes. These aggregates trigger lysosomal leakage due to their physical features and release the contents into the cytosol through a mechanism mediated by lysosomal cysteine protease, cathepsin B (CTSB), augmenting the NLRP3 complex activation [18, 49, 50]. In agreement with this model, crystalline aggregates like silica are the main activator of IL-1β secretion via the inflammasome complex. Furthermore, macrophages that lack cathepsin-B demonstrate a moderate or undetectable role in the inflammasome activation [51]. However, Ca-074-Me, a multiple cathepsin inhibitor, has been shown to induce NLRP3 inflammasome activation through an off-target effect [52]. More recently, reports have shown that oxidative stresses especially ROS in CTSB up-regulation may induce inflammasome formation [49, 53]. It has been shown that the chemotherapy agents gemcitabine and 5-fluorouracil (5-FU) that cause an excess of ROS induce CTSB release, activates the NLRP3 complex [54] though the exact function of CTSB in NLRP3 activation needs further investigation.

## Role of the NLRP3 inflammasome in cancer

Inflammation and persistent infection may contribute to various human malignancies [55, 56]. Evidence has accrued on the role that inflammation has in cancer initiation, development, progression, angiogenesis and invasion [57, 58]. Inflammation may induce an immune response involving T cells, B cells, NK cells, DC, macrophages and neutrophils [59, 60]. However, the exact role of the immune system in up-regulation and modification of self-antigens in tumorigenesis remains unclear. As noted above, inflammasomes are the multi-protein platform in the innate immune system that induce procaspase-1 activation and inflammatory cytokines maturation such as IL-1β and IL-18 [61]. Over-expression of IL-1β can influence several autoimmune diseases and may result in carcinogenesis [62]. Several inflammasomes including, NLRP3, NLRP6, NLRC4, NLRP1 and AIM2 may have a pathogenic role in tumorigenesis by their modulation of innate and adaptive immunity, apoptosis, differentiation, and the gut microbiota [63]. There is now data suggesting that NLRP3 inflammasome polymorphisms are related to different malignancies such as colon cancer and melanoma (Table 1) [64]. The precise clinical function of NLRP3 in the role of the initiation and promotion of differing neoplasms also highlights the therapeutic potential of inflammasomes, and as prognostic markers.

**Table 1:** Role of NLRP3 inflammasome activation or suppression in cancer development

Type of cancer	Source of experimental evidence	Outcome	Suggested mechanism	References
HNSCC	- HNSCC cell lines (A253) -Oral cancer tissue	Activation of NLRP3 inflmmasome closely associated with survival and invasiveness of HNSCC	Activation of IL-1β	[99]
	-HNSCC tissue - HNSCC cell lines(CAL27, SCC9, SCC25, and FaDu) - transgenic mouse HNSCC model	NLRP3 inflammasome related with the tumorgenesis and CSCs markers self-renewal activation	-overexpression of CSCs markers (BMI1, ALDH1 and CD44)	[100]
GBM	-U87 and GL261 xenograft mouse GBM model	NLRP3 inflammasome involved in resistance to radiotherapy	-regulation of numerous aging-related genes in hippocampus	[150]
OSCC	- OSC cells lines (WS UHN6 and C AL27) -NLRP3^−/−^ and Caspase1^−/−^ mice -OSCC tissue	NLRP3 inflammasome increased resistance of OSCC to 5-FU	Promotion of the IL-1β production	[106]
BC	- BC cell lines(LLC and E0771) -C57BL/6 mice	tumor-infiltrating regulation of NLRP3 strongly linked with tumor invasiveness, migration and outcome	IL-1β secretion and S1PR1 signaling	[123]
GC	-GC tissue -GC cell lines (SGC-7901, BGC-823, HGC-27 and AGS) -normal gastric epithelial cell line (GES-1)	NLRP3 inflammasome stimulates epithelial cells proliferation and GC carcinogenesis	-IL-1 β secretion -enhance cyclin-D1 transcription	[77]
CAC	-NLRP3^−/−^, Pycard^−/−^ and Caspase1^−/−^ mice	Mice with inflammasome compartment deficiency were extremely susceptible to AOM/DSS- induced colitis	-reduction in IL-18	[67]
	-NLRP3^−/−^ mouse	NLRP3^−/−^ mouse is more susceptible to acute and recurrence CAC	-increasing pro–IL-1β and IL-18 secretion	[85]
	-NLRP3^−/−^ and Caspase1^−/−^ mice	NLRP3^−/−^ and Caspase1^−/−^ mice were more susceptible to AOM/DSS-induced inflammation and increased tumor burdens	- NLRP3 inflammasome deficiency lead to reduction in secretion and activation of the tumor IFN-γ and STAT1	[86]
	-NLRP3^−/−^, ASC^−/−^, Caspase1^−/−^, cathepsin B^−/−^ or cathepsin L^−/−^mice-	NLRP deficient mice were significantly protected from colitis	IL-1β secretion was abrogated in macrophages without NLRP3, ASC or Caspase-1	[165]
CRC	-CRC and adjacent normal tissue	NLRP3 gene variation are correlated with worse survival	-elevatating IL-1β and IL-6 levels	[89]
CRC metastatic in liver	-Inflammasome components ^−/−^ mouse	NLRP3 inflammasome inhibits liver CRC metastatic growth	-enhancing NK cell tumoricidal action	[91]
Fibrosarcoma	-NLRP3^−/−^ mouse model	NLRP3-deficient mice were less resistant to tumor formation	-NLRP3 suppressed NK cell	[116]
Melanoma	-Human melanoma cell lines (A375) -mouse melanoma cell lines (B16F10)	Inhibition of NLRP3 inflmmasome blocked melanoma migration	-inhibition of NLRP3 inflmmasome suppressed secretion of cytokines IL-1β and IL-18	[139]
Cervical Cancer	-HPV^+^ and adjacent normal tissue	NLRP3 polymorphism related with a lower risk of HPV infection	-innate immune anti-viral response - obliteration of virus persistence and viral elimination	[144]
Lung cancer	-human alveolar epithelial adenocarcinoma cell line (A549)	NLRP3 inflmmasome regulate the proliferation and metastasis of lung cancer	-promoting phosphorylation of Akt, ERK1/2, and CREB -enhancing the expression of Snail -decrement of E-cadherin expression	[112]
HCC	-HCC tissues and adjacent normal tissues	-Down regulation of all of the NLRP3 inflammasome elements associated with HCC occurrence, advanced tumor stages and poor differentiation	NR	[96]

*Abbreviations: ASC* apoptosis-associated speck-like protein, *AOM/DSS* Azoxymethane/dextran sodium sulphate, *BC* Breast cancer, *CAC* colitis-associated colorectal cancer, *CSCs* cancer stem cells, *CRC* Colorectal cancer, *GC* Gastric cancer, *GBM* Glioblastoma; *HCC* hepatocellular carcinoma, *HNSCC* head and neck squamous cell carcinoma in humans, *NK* Natural killer cell, *OSCC* oral squamous cell carcinoma, *5-FU* 5-fluorouracil, *NR* not reported

### Role of the NLRP3 inflammasome in colonic epithelium homeostasis

The inflammasome complex is a vital homeostatic component in the intestinal epithelium. Experimental mice models have highlighted the clinical features of the inflammasome that have been related to human disorders [65]. In one model, oral administration of dextran sodium sulfate (DSS) gave rise to an increased mortality rate and loss of body weight through the toxic effects in colon epithelium [66], and NLRP3 deficient mice showed an increased mortality, diarrhea and rectal bleeding. The enhanced intestinal inflammation in NLRP3-deficient mice induced by DSS was due to increased colonic permeability, with pathogens lodging in the liver and lymph nodes [67]. In another study ASC and caspase-1 deficient mice showed increased histopathological changes that were associated with death in both chronic and acute inflammation [68]. Others reported that in both NLRP3 and caspase-1 deficient mice, the proliferation of gastrointestinal epithelial cells were reduced [67]. The increased permeability in NLRP3-deficient mice was linked to decreased antimicrobial efficacy and a reduction in colonic defensins production [69].

### NLRP3 inflammasome in gastrointestinal malignancies

#### NLRP3 in gastric cancer

Gastric cancer (GC) is the fourth most prevalent malignancy and is a global health problem. Persistent infection with the bacterium *Helicobacter pylori* (*H. pylori*) leads to the development of gastric and extragastric disorders [70]. It has been found that he NLRP3 may be involved in the pathophysiology of *H.pylori* infection and IL-1β production [71]; NOD1 protein is significantly increased and followed by an elevation of inflammation in gastric neoplasms induced by *H.pylori* [72]. In addition, NOD2 regulate the microbiota and maintenance of colon tumors [73]. The NLRP3 inflammasome enhances cell differentiation in gastric cancer by engaging cyclin-D1 as well as inducing IL-1β production. IL-1β binds to its receptor and activates NF-κB that initiates JNK signalling causing proliferation, invasion and cancer development [74]. *H.pylori* infection leading to gastric chronic inflammation and mediation of the inflammatory cytokines such as IL-6, IL-1β, tumor necrosis factor alpha (TNF-α), and macrophages [75], may trigger gastric cell proliferation and carcinogenesis [76].

Therefore, NLRP3 by a combination of dependent and independent inflammasome pathways may increase proliferation of gastric cancer cells and GC development. NLRP3 down regulation may modify the mechanism of GC progress [77]. A number mechanisms exist for the loss of NLRP3 expression including the aryl hydrocarbon receptor (AhR), Dopamine D1 receptor (DRD1) and GPBAR1 (G-protein coupled bile acid receptor 1). The AhR mechanism prevents binding of the xenobiotic response element to the NLRP3 inflammasome transcription factor and DRD1 acts via E3 ubiquitin ligase membrane associated ring-CH-type finger 7 (MARCH7) that can lead to NLRP3 inflammasome ubiquitination and autophagy reaction [78–80]. Variations in IL-1β have been shown to be related to GC susceptibility and induced liver tumorigenesis [81]. Li et al. reported that microRNA (MiR)-22 is an essential modulator in the stomach by down regulation of NLRP3 in liver mucosa cells and macrophages, and *H. pylori* infection significantly inhibited miR-22 expression whilst promoting NLRP3 expression. This suggests that miR-22 has a significant role in the inhibition of NLRP3 inflammasome expression. In addition, it has been demonstrated that the miR-22 is able to inactivate gastric cell proliferation and carcinogenesis induced by *H.pylori* [77]; however, the molecular pathways between the NLRP3 inflammasome and gastric tumorigenesis require further elucidation.

#### NLRP3 in colitis associated tumorigenesis

Colorectal cancer (CRC) is the third most common cause of cancer mortality in the United States [82]. Colonic inflammation that occurs in response to damage and pathogens can increase CRC susceptibility [83]. The mechanism of NLRP3 inflammasome in tumorigenesis of colorectal cancer suggested that the antitumor effect of IL-18 prevents tumors development as well as inhibiting angiogenesis and may induce epithelial cell recovery [68, 84]. NLRP3 and caspase-1 deficient models induced by azoxymethane (AOM)/DSS showed significant decreases in IL-18 in the intestine [67, 85, 86]. Oral administration of DSS gives rise to multiple clinical and histopathological features associated with ulcerative colitis in humans including bloody diarrhea, weight loss, crypt and epithelial cell edema and injury, as well as leukocyte infiltration [87]. In addition, administration of recombinant IL-18 in caspase-1 deficient animal models treated with AOM/DSS significantly prevented tumor development [86]. Conversely, in the IL-18 deficient AOM/DSS murine model the cancer burden increased mirroring NLRP3 and caspase-1 deficient mice [88]. These data indicate the important role of IL-18 secretion through NLRP3 that protects colitis from malignant transformation, and promotes enterocyte differentiation and intestinal epithelium integrity [87]. Furthermore, in the colitis remission phase IL-18 can reduce cell proliferation in the intestinal epithelium at the tumor zone [86]. IFN-γ was shown to be significantly decreased in AOM/DSS mice models that lacked NLRP3 and caspase-1, suggesting that IFNγ could increase colon cell proliferation in primary grade DSS-induced colitis [87]. Ungerback et al. showed that variations in tumor necrosis factor alpha–induced protein 3(TNFAIP3), NLRP3 and NFκB genes were related to CRC susceptibility [89]. Grace et al. described that caspase-1 deficient mice showed severe tumorigenesis as well as STAT1 and IL-18 reduction compared to the NLRP3-deficient model [90]. It has also been reported that the NLRP3 Inflammasome inhibits CRC metastatic growth in the liver through enhancing NK cell tumoricidal function that was mediated by IL-18, independent of IFN- γ, as knockout mice for the NLRP3 inflammasome show increased liver CRC metastases [91].

#### NLRP3 in hepatocellular carcinoma

Hepatocellular carcinoma (HCC) is the fifth most prevalent neoplasm and it is the third most common cause of cancer death in the world [92]. The hepatic parenchymal cell stroma is associated with the invasiveness of hepatocellular cancer [93]. A substantial body of evidence has confirmed the role of the NLRP3 inflammasome in liver failure and liver disease [94, 95]. Within HCC, the NLRP3 inflammasome molecular platform components are lost or significantly reduced compared to normal liver, and its down-regulation is significantly associated with advanced clinical stages and poorer pathological differentiation [96, 97]. Conversely, targeting the NLRP3 inflammasome pharmacologically may repress proliferation and metastasis of HCC, suggesting that this could be a therapeutic strategy and indicates that understanding the exact mechanisms of action of the inflammasome in HCC tumor proliferation, aggression and metastasis is required [98].

### NLRP3 inflammasome in non-gastrointestinal malignancy

#### NLRP3 in head and neck cancer

Head and neck squamous cell carcinoma (HNSCC) is closely related to chronic inflammation, and elevated NLRP3 inflammasome expression in HNSCC tissue has been shown and the degree of expression has been associated with disease prognosis. HNSCC can induce the production of active IL-1β through NLRP3 inflammasome pathways, and inhibition of the NLRP3 inflammasome pathway was suggested to be a promising approach for decreasing tumour cell invasion and survival [99]. More recently, NLRP3 inflammasome activation has been show to activate cancer stem cells (CSCs) leading to self-renewal and acceleration of HNSCC progression, thus NLRP3 inflammasome inhibition could decrease the CSCs population in HNSCC with a consequent improvement in prognosis [100].

Oral cancer is the sixth most prevalent malignancy in the world and oral squamous cell carcinoma (OSCC) accounts for approximately 90% of all oral malignancies [101, 102]. Whilst the possible molecular mechanisms in OSCC are being determined, the definitive reason for the initiation and development of OSCC is still not clear [103]. Inflammation as a major cause of tumorigenesis that is linked with genetic and epigenetic changes and can induce OSCC [104]. It has been reported that NLRP3 inflammasome components are up-regulated in animal OSCC models and OSCC patients. 5-FU is a chemotherapeutic agent that is used for the treatment of solid malignancies such as OSCC, but due to a resistance to therapy it has a narrow spectrum of clinical use [105]. NLRP3 inflammasome down-regulation has the potential to be a new therapeutic approach [100], as it has been demonstrated that NLRP3 inflammasomes are elevated in 5-FU chemoresistance both in vitro and in vivo in OSCC cells; therefore, targeting the NLRP3 inflammasome/ROS/IL-1β signaling pathways may improve chemotherapy with 5-FU [106].

#### NLRP3 in lung cancer

Lung cancer has been shown to be initiated by a number of differing environment exposures pathogens [107], and it is well recognised that chronic inflammation is a critical factor for lung tumour progression [108]. Asbestos induces NLRP3 inflammasome activation in mesothelial cells leading to an inflammatory response and eventually cancer initiation and progression [109]. Animal work has shown high NLRP3 mRNA expression levels in lung and spleen [110], with the highest NLRP3 mRNA expression being found in alveolar macrophages [110, 111]. Wang et al. reported that IL-18 and IL-1β secretion was elevated due to NLRP3 inflammasome activation in the lung adenocarcinoma cell line A549, and they suggested that a combination of IL-18 and IL-1 β cytokines may have therapeutic potential [112]. Other studies have shown NLRP3 activation following allergen exposure enhanced N6-etheno- ATP (eATP) in bronchoalveolar lavage (BAL) fluid resulting in an elevation of IL-1β in asthma [113]. TNF-α has an effective role in the survival from malignant mesothelioma by inhibition of mesothelial proliferation, diminution of asbestos damage and induction of NF-κB [114]. This subsequently leads to the formation of an inflammasome complex and the production of IL-1β that is important for malignant mesothelioma progression [115]. In NLRP3 deficient mice, lung tumour cells were decreased compared to control animals [116]. Nanoparticles (NP) such as silica and asbestos may result in the overexpression of NLRP3 inflammasomes, and the secretion of caspase-1 and IL1β in the in vivo model of lung cancer [117]. NP inhalation could increase chronic pulmonary disorder susceptibility; however, the definitive function of NLRP3 in chronic obstructive pulmonary disease (COPD) and asthma is unclear [113].

#### NLRP3 in breast cancer

Breast malignancy is the fifth cause of mortality among women globally [118]. There is no direct evidence for NLRP3 inflammasomes in breast cancer; however, indirect evidence implicates a role of inflammasome activation in breast tumour development through IL-1β [119]. IL-1β is up regulated in breast neoplasm initiation and development [120] and also IL-1R and IL-1β variations have been related to breast tumorigenesis [121]. The fibroblast growth factor receptor (FGFR) 1 in the breast malignancy animal model leads to mammary carcinogenesis and is related to IL-1β secretion [119]. Tumour-associated macrophages (TAMs), among other tumor-infiltrating immune cells, play a major role in tumor lymphangiogenesis and propagation. Inflammasome activation followed by IL-1β and sphingolipid sphingosine-1-phosphate (S1P) signaling production in TAMs facilitates a favorable microenvironment for mammary carcinoma development [122, 123]. The S1P signaling is involved in several cellular biological pathway and possibly regulates growth, proliferation, development, and survival [124]. It has been shown that amplification of the NLRP3 inflammasome components was reduced in S1PR1-deficient TAMs suggesting that NLRP3 regulation in TAMs was associated with lymph node metastasis and prognosis [123].

#### NLRP3 in prostate cancer

Prostate malignancy is a common cause of cancer mortality among males in western countries [125]. Pathogens, destructive signals and stresses are the usual stimulatory factors for NLRP3 Inflammasome activation in prostate tissue [126], but other mediators such as uric acid, infections and urine crystals can induce prostate gland (PG) injury that lead to up regulation of proinflammatory cytokines through the activated inflammasome in the PG and lead to cancer progression [127].

Animal models have shown over-expression of inflammasome protein in prostatic inflammation through chemical stimulators inducing caspase-1 and IL-1훽 activation through NRLP1 inflammasome up regulation in PG [128]. NLRP3 deficient mice show reduced cancer invasion and tumorigenesis through a reduction in NK cell proliferation and CXCL9 chemokine secretion. The mouse models using an oxalate diet promote kidney damage and result in NLRP3 activation [129]; however, the data is inconsistent on whether NLRP3 deficiency is a risk factor for tumor formation due to the inflammatory response [85]. An association between the inflammatory response and autophagy has been shown with a malfunction in autophagy inducing NLRP3 activation resulting in a decrease in IL-1β [130]. This process is linked with the endoplasmic reticulum stress that results in activation of the NLRP3 complex leading to prostate malignancy progression [131]. It is also reported that in the prostate cell lines (BPH-1 and PC-3) exposed to hypoxia, NF-κB is over-expressed leading to NLRP3 and AIM2 inflammasome activation [132]. However, others have reported conflicting results that indicated there was no significant difference in NLRP3 inflammasome expression level in all of the prostate cancer stages examined [133].

#### NLRP3 in skin cancer

Skin neoplasms, melanoma and non-melanoma malignancies, are the most prevalent types of cancers in white populations [134]. Melanoma research demonstrated that the development of cancer cells was inhibited by reduced inflammasome and IL-1β expression [135]. Recent evidence suggested that NLRP3 inflammasome up regulation may aggravate inflammatory responses in skin neoplasms. Mice models with NLRP3, caspase-1 and ASC adaptor deficiencies show protection against cancer progression [110, 136]. Melanoma expresses the inflammatory characteristics depending on the grade of tumor. In the first grade IL-1 receptor and co-stimulatory molecules are highly expressed. In the second grade, IL-1R is expressed and in the third progressive stage the NLRP3 inflammasome is active constitutively [137], a correlation has been shown between the NLRP1 and NLRP3 variations and melanoma risk, with the greatest correlation between NLRP1 and nodular melanoma [138]. In accord with this data it has been shown that NLRP3 down regulation and decreased IL-1β and IL18 secretion reduced metastatic melanoma by thymoquinone therapy in a mouse model [139]. Evaluation of the role of the NLRP3 inflammasome in the immune response by using DC vaccination against the melanoma cells showed that vaccination of NLRP3 deficient mice who received a subcutaneous injection of poorly immunogenic melanoma cells resulted in a 4-fold promotion in overall survival compared to control animals [140]. Others have reported that NLRP1 can activate caspase 2 and − 9 in neoplasm cells resulting in tumorigenesis, but NLRP3 did not appear to be tumorigenic [141].

#### NLRP3 in cervical cancer

Cervical malignancy is the second most prevalent neoplasm in females globally [142]. Recent reports showed that human papillomavirus (HPV) is able to trigger abnormal cell growth in the cervix via inflammation [143]. Pontillo et al. reported that a variant in the NLRP3 gene, rs10754558, was associated with HPV resistance and showed that there was a statistically significant relationship between rs10754558 and cervical cancer development [144]. Others have reported that CD200 (a membrane glycoprotein belongs to immune globulin super family) can suppress NLRP3 and TLR4-NF-κB pathways in LPS-induced human cervical cancer cell lines [145].

#### NLRP3 in central nervous system tumors

Brain and central nervous system (CNS) malignancies are uncommon neoplasms associated with differing pathological causes, molecular pathways and immunological responses [146]. The innate immunity has an important role in cancer metastasis in the CNS [147]. Like other cancers, numerous elements are expressed in the brain tissue including TLR and NLR, which can result in pro-inflammatory responses and activate inflammasome complex formation for tumorigenesis. In this regard, NLRP3 can decrease NK cell activation and lead to tumor invasion [116]. Also, it has been shown that IL-1β is aberrantly expressed in glioblastoma cells as a result of NLRP3 inflammasome activation [148]. As a result, NLRP3 may be important in carcinogenesis and its elevation may be used as a predictive biomarker in future therapeutic strategies [149]. In an experimental model of glioblastoma, preventing NLRP3 expression decreased cancer development and enhanced survival rate in the mice undergo ionizing radiation (IR) therapy, with NLRP3 being a bridge between brain ageing/glioma development and IR therapy [150]. Therefore, reducing NLRP3 gene expression may be a future therapeutic target for gliomas though further clarification of the molecular mechanisms is needed.

### NLRP3 and cancer in human studies

Knowledge about inflammasomes and cancer in human studies is preliminary and scarce.

Recently, inflammasome activation as a major cause of inflammation was suggested in human adamantinomatous craniopharyngioma (ACP), a rare tumor of children occurring in the sella region. The expression of numerous genes regulating core inflammasome components including *NLRP1*, *NLRP3*, *NLRC4*, *CASP1* and *PYCARD* was increased by a maximum of 6.4, 4.8, 4.8, 5 and 4 fold, respectively [151]. This result could have therapeutic implications. It has been shown that suppression of inflammasome activation by IL1 inhibitors such as anakinra and canakunimab had significant effects in various neuroinflammatory and autoinflammatory diseases [152–154]. Therefore, these inhibitors may have therapeutic potential in cancer therapy that needs further assessment.

Takano and colleagues demonstrated higher expression of proteins related to inflammasome complex (e.g. NLRP3, ASC, IL-1β, IL-18 and caspase-1) in patients with oropharyngeal squamous cell carcinoma (SCC) compared to controls irrespective of their HPV infection status. Since the over-expression of inflammasome-related proteins in oropharyngeal SCC is independent to HPV infection, this indicates the inflammasomes may play a major role in promoting antitumor immunity [155]. Recently, up-regulation of NAIP, NLRP3, NLRP4 and NLRP9 were found in patients with bladder cancer versus normal controls [156]. Moreover, it has been observed that pro-inflammatory cytokines including IL1β and NLRP3 were significantly up-regulated in visceral adipose tissue versus subcutaneous adipose tissue in cancer patients. Expression levels of IL1β and NLRP3 were directly correlated with mean diameter of adipocytes (μm) in males, but not in females [157] and it has been suggested that the NLRP3 inflammasome could a novel biomarker for obesity-related metabolic diseases [158, 159], as the high-amplification of IL1β and NLRP3 may be connected to pathophysiological abnormalities in visceral adipose tissue.

The level of NLRP3 was considerably augmented in cancerous plasmacytoid dendritic cells (pDCs) isolated from human lung samples of patients suffered from non-small cell lung cancer than samples from normal controls. Notably, the triggering of tumor-associated pDCs with the NLRP3 activator caused elevated IL-1β levels [160].

Several genetic studies have investigated the relevance of the NLR signalling pathway in different human cancers. For instance, patients with pancreatic cancer have the rs35829419-NLRP3 polymorphism (other name, Q705K) at a greater frequency than non-cancer individuals. Q705K (glutamine to lysine) may lead to over enzymatic cleavage of pro-IL-1β to its active form [161]. Wang et al. genotyped selected inflammasome compartment-SNP’s rs16944 in *IL-1β*, rs1946518 in *IL-18*, Q705K in *NLRP3*, and rs2043211 in *CARD8* among 383 acute myeloid leukemia (AML) patients and 300 pairs control. Results showed that only variations of IL-18 and IL-1β were associated with the clinical characteristics and decreased survival of AML patients [162]. Similar results were also observed in chronic myeloid leukemia (CML) patients in which genotyping demonstrated that genetic polymorphisms of *IL-1β*-rs16944, *IL-18*-rs1946518, and *CARD8-*rs2043211 were associated with the pathophysiological characteristics and treatment of CML patients. These variations may be applied as a novel prognostic and therapeutic markers for leukaemia, which requires further evaluation in the future studies [163].

Another study evaluated the association of the genetic polymorphisms of *NLRP3* (Q705K and rs10733113), *CARD-*8 (rs2043211), and *NLRP1* (rs6502867 and rs12150220) in Swedish patients with sporadic malignant melanoma (MM). Swedish males carrying rs35829419A- NLRP3 are more susceptible to sporadic MM. In particular the presence of nodular melanoma (NM) was associated with NLRP3-rs35829419 and NLRP1-rs12150220. Furthermore, the NLRP1-rs12150220 was 1.8 times more prevalent in fair-skinned female patients (CI:1.04–3.3) [138]. However, these results for melanoma association were not found in a Brazilian cohort, even though the frequencies of commonly selected SNPs (Q705K, NLRP1-rs12150220, and *CARD-*8-rs2043211) are similar to the Swedish population. [164] In a case-control gene expression study, variants in *CARD8* (rs11672725), *NLRP3*)rs10754558(*NLRP3*)rs4612666(*NLRP12*)rs199475867 (and *NLRX1*)rs10790286 (were significantly associated with GC. Multivariate regression analysis demonstrated that *CARD8*-rs11672725 and *NLRP12*-rs2866112 were strong risk factor for GC and *H. pylori* infection (OR = 4.8, 95% CI: 1.4–16.6; and OR = 2.1, 95%CI: 1.2–3.7), respectively [164].

## Conclusion

Inflammation induced through microbial or danger signals affects all stages of tumor development and the pro-inflammatory cytokines, IL-1β and IL-6, are important mediators for inflammation-induced tumorigenesis. The NLRP3 inflammasome is an intracellular complex that regulates the innate immune activity through modulation of the production of pro-inflammatory cytokines.

There is increasing attention directed toward identifying the role of the NLRP3 inflammasome in differing tumor types, and the activation of inflammasomes in tumor formation, development and invasion remains controversial and conflicting. Research using AOM/DSS-induced colitis and colon cancer in knockout animal models suggested a protective role of inflammasome components against carcinogenesis. Conversely, lung cancer, melanoma, breast cancer and HNSCC, demonstrated that NLRP3 inflammasomes, IL-1β and IL-18 promote tumor growth, proliferation, invasion and metastasis. Furthermore, in glioblastoma and oral squamous cell carcinoma, NLRP3 inflammasomes are associated with chemoradioresistance. In most of the reports cited, the evidence suggesting that the NLRP3 inflammasome is contributing to cancer progression in vivo remains preliminary and requires further confirmation. It has been suggested that inflammasome activation in tumors specifically depend on the tissue-context to whether inhibition or activation of tumorigenesis results. Further studies are required to address the molecular mechanisms behind the production, activation, and modulation of inflammasomes and to determine their potential therapeutic role in human malignancy.
